# From laboratory to industrial storage – Translating volatile organic compounds into markers for assessing garlic storage quality

**DOI:** 10.1016/j.postharvbio.2022.111976

**Published:** 2022-09

**Authors:** Richard A. Ludlow, Gareth Evans, Michael Graz, Gracia Marti, Puri Castillo Martínez, Hilary J. Rogers, Carsten T. Müller

**Affiliations:** a School of Biosciences, Cardiff University, Sir Martin Evans Building, Cardiff CF10 3AX, UK; b Neem Biotech, Units G&H Roseheyworth Business Park, Abertillery NP13 1SX, UK; c Coopaman S.C.L., Ctra de las Peñas, km 1’6, 02006 Albacete, Spain

**Keywords:** *Allium sativum*, VOC, GC-MS, Alliinase, Storage

## Abstract

Garlic (*Allium sativum* L.) has long been grown for its culinary and health-promoting qualities. The seasonal nature of garlic cropping requires that bulbs be stored for many months after harvest to ensure a year-round supply. During this time, quality is known to deteriorate, and efforts have been made to improve the longevity of stored bulbs. Cold temperatures within the stores prolong shelf life, but fine temperature control is needed to avoid freezing damage or cold induced stress. Here, quality traits (alliinase activity, firmness, and water content) are measured in response to a 96 h − 5 °C cold stress, to simulate the effect of non-isothermic temperature control in a − 1.5 °C warehouse. Volatile organic compounds (VOCs) are measured by thermal desorption gas chromatography time of flight mass spectrometry to identify markers of non-isothermic storage in garlic. 129 compounds were putatively identified and four (L-lactic acid, 2,6-dimethylhetpadecane, 4-methyldodecane, and methylcyclopentane) showed high predictive accuracy for cold stress. VOCs were also sampled directly from a cold storage facility and the whole profile discriminated between sampling time points. Five VOCS were highly predictive for storage time in the warehouse but were different to VOCs previously shown to discriminate between storage times in a laboratory setting. This indicates the need for realistic warehouse experiments to test quality markers.

## Highlights

• Four VOC markers can detect breaches in a garlic cold storage regime.
• VOCs provide a tool for monitoring garlic quality in the warehouse.
• VOCs discriminate storage time of garlic bulbs in a warehouse.
• Time discrimination based on VOCs was independent from garlic variety.

## 1 Introduction

Over 30 million tonnes of garlic (*Allium sativum* L*.*) were produced worldwide in 2019, making it an important commercial crop. Garlic is consumed not only for its culinary uses, but also for its health promoting properties. Garlic powder intake reduces the risk of cardiovascular disease (Kwak et al., 2014, El-Saber Batiha et al., 2020), and garlic extracts have wide-spectrum antimicrobial properties (El-Saber Batiha et al., 2020). Intake of garlic has also been associated with reduction of colorectal cancer (Farhat et al., 2021) and garlic phytochemicals have potential for cancer treatments (Zhang et al., 2020). Garlic bulbs produce a wide range of phytochemicals, many of which are sulphur-containing compounds, including ajoenes and thiosulfinates such as allicin. Allicin is derived from the enzymatic action of alliinase on the cysteine sulphoxide alliin, which only occurs when cells are ruptured, due to separation of enzyme and substrate within intact cells (Ankri and Mirelman, 1999). Alliin is non-volatile and stable, while allicin is highly reactive and can further transform into volatile compounds such as diallyl disulphide, diallyl sulphide and diallyl trisulphide (Block, 1992). Most garlic supplements consist of enteric-coated tablets containing alliinase and alliin, due to this instability. These tablets can yield lower amounts of allicin than expected, either through impaired alliinase activity, or slow tablet disintegration (Lawson and Wang, 2001). As such, garlic with high alliinase activity is essential for the manufacture of garlic supplements. Alliinase constitutes up to 12% of garlic clove protein (Ellmore and Feldberg, 1994), and can be readily extracted from garlic, however it is not very stable due to the presence of free thiol groups which can become oxidised (Krest and Keusgen, 1999, Janská et al., 2021). Thus, for biotechnological extraction it is preferable to use fresh garlic bulbs.

The seasonal nature of garlic cropping requires bulbs to be stored for prolonged periods of time to facilitate year-round supply, during which time quality falls (Block, 2010). The storage temperature influences key quality traits over time in storage, including sprouting, weight loss and disease incidence (Rahim and Fordham, 1988, Mann and Lewis, 1956). Storage conditions also affect biochemical quality traits, however there is less research into effects of storage on alliinase activity (Martins et al., 2016, Ichikawa et al., 2006, Kodera et al., 2002, Ludlow et al., 2021). The control of sprouting is temperature dependent in *Alliums*, with higher storage temperatures (>25 °C) and low temperatures (< 0 °C) exhibiting low rates of sprouting over long-term storage by maintaining dormancy in garlic and onion (Cantwell et al., 2003, Ichikawa et al., 2006, Ramin, 1999). Postharvest spoilage organisms such as *Aspergillus niger*, *A. alliaceus* and *Pantoea ananatis* are prevalent on *Allium* bulbs and in the soil of fields where *Alliums* have been grown (Varga et al., 2004, Abd-Alla et al., 2006, Walcott et al., 2002). Disease control in stored garlic is largely achieved by managing temperature and humidity (Block, 2010, Tian and Bertolini, 1995, Llamas et al., 2013). Low storage temperatures, typically below 0 °C, show good long-term control of a number of postharvest diseases of garlic, both through inhibiting fungal pathogenicity (Llamas et al., 2013, Lee and Magan, 2010, Bertolini and Tian, 1996) and maintaining garlic clove dormancy (Brewester, 1994). Indeed, accurate storage temperatures can be extremely important in managing disease in garlic, with for instance the control of *Penicillium hirsutum* being significantly greater at − 4 °C than − 2 °C (Tian and Bertolini, 1995). A relative humidity of 60–70% RH is considered optimal for garlic storage, as higher RH favours fungal growth and lower RH causes excessive moisture loss from the bulbs (Shiina et al., 2004, Brewester, 1994).

Garlic is also subject to cold damage from freezing due to either mechanical injury to cells during ice crystal formation or from cell dehydration. Water is drawn out of solution as it crystallises, concentrating the solutes in the cell (Pearce, 2001, Muldrew and McGann, 1990, Enüstün et al., 1978). Any freezing damage reduces the quality of garlic significantly and often leads to total loss of marketability. Freezing damage is apparent visually upon thawing (James et al., 2009), and results in a failure of the compartmentalisation of alliinase and alliin, detectable as a strong odour. Garlic is reported to have a freezing range of − 2.6 to − 3.2 °C, with a mean of − 2.7 °C (James et al., 2009). However, garlic is often stored commercially below these temperatures with no ill effect. Indeed, storing garlic at − 4 °C caused no noticeable deleterious effects (Tian and Bertolini, 1995). The absolute limit of temperature tolerance in garlic is related to the duration of exposure. Peeled garlic cloves were able to withstand temperatures as low as − 9 °C for just under 3 days, and for a week at − 6 °C without freezing (James et al., 2009). At an industrial storage level, therefore, the concern instead is that lower than planned storage temperatures that do not cause freezing damage may still affect alliinase activity or other bulb quality characteristics but remain undetected.

Analysis of volatile organic compounds (VOCs) can be used to assess postharvest quality in a range of different crops (Tiwari et al., 2020) including garlic (Ludlow et al., 2021). Different methodologies have been applied for VOC collection, detection and identification (Tiwari et al., 2020) however some such as SPME (solid phase microextraction) or direct headspace-based methods can be difficult to translate into a commercial storage setting for monitoring quality. Collection of VOCs onto TENAX thermal desorption tubes requires no complex equipment on-site thus allowing remote and passive sampling in situ. This approach could therefore be used to measure average VOC profiles within a storage facility, and TENAX tubes show good reproducibility with garlic VOCs (Pino, 1992). The VOCs are stable for several weeks once adsorbed onto the tube, affording time for the tubes to be sent to the lab for analysis. This can be performed in a matter of days from the completion of sampling, yielding results in a meaningful time frame for a crop which is stored for several months. Furthermore, modern thermal desorption units (e.g TD100 Markes International) allow recollection of samples from a split injection, which facilitates repeated injections with different split ratios and allows for the detection of compounds at < 10 ng/kg level whilst also allowing relative concentrations of the highly abundant compounds to be measured without overloading the detector (Kim et al., 2013, Woolfenden, 2010).

Here the aims were to assess independently two key problems facing garlic storage, primarily focused on the biotechnological uses of the bulbs. Firstly, we aimed to assess whether VOC analysis is a useful indicator of poor temperature control during storage resulting in periods of excessive cold by comparing bulbs maintained isothermally to bulbs that had been exposed to a cold stress treatment. Secondly, we aimed to assess whether the collection of VOCs onto thermal desorption tubes for monitoring changes in quality during postharvest storage could be transferred to a warehouse setting.

## 2 Materials and Methods

### 2.1 Plant material

Category I quality garlic bulbs *(Allium sativum cv.* Morado and Blanco*)*, 60–65 mm in diameter, were provided by Coopaman, 45 Las Pedroñeras, Cuenca, Spain and were previously inspected for disease and damage. After harvest, bulbs were dried and processed at Coopaman.

### 2.2 Cold Stress

Bulbs of garlic *cv.* Morado were shipped to Cardiff after drying but before cold storage and were cold stored at − 1.5 °C ± 0.6 °C in a custom-built chilled cabinet for 5.5 m, after which half were transferred to a separate cabinet and cold stressed for 96 h at − 5 °C ± 0.05 °C. The two cabinets were of similar size and specifications. This temperature was selected to be above that resulting in freezing damage (James et al., 2009) but below standard commercial storage temperature. After the − 5 °C treatment, the cold stressed bulbs were returned to the − 1.5 °C incubator and stored for the remainder of the 6 month storage period with the control samples. All three replicates for each treatment were stored in the same cabinet.

### 2.3 Physiological measurements of stored garlic

Clove firmness was measured with a Bishop model FT 327 Fruit pressure Tester (Facchini SRL, Italy), using a 0.5 cm^2^ penetrometer tip. The outer sheath of the clove and the upper epidermis of the clove were removed with the peeler provided with the penetrometer. The pressure probe was slowly pushed into the peeled area at a uniform rate, midway from the basal plate to the tip of the bulb, angled directly towards the centre of the clove. The maximum force exerted to push the probe 5 mm into the clove was reported in kilogram force (kgf), as per industry standards, where 1 kgf = 9.807 N. Cold stored garlic was returned to room temperature overnight before testing. Bulb moisture content was measured as described previously (Ludlow et al., 2021).

### 2.4 Alliinase enzyme extraction, protein concentration and alliinase activity assay

Alliinase was extracted from three bulbs of garlic that were broken apart into their constituent cloves to yield 50 g of peeled, disease free cloves. The extraction, followed by measurement of protein concentration and alliinase activity were performed as described previously (Ludlow et al., 2021).

### 2.5 VOC collection and TD-GC-ToF-MS Analysis

Lab based VOC collection from three bulbs, selected at random, was as described previously (Ludlow et al., 2021). Three independent groups of three bulbs were collected to provide three replicates, as well as blanks consisting of air sampled in an identical fashion from the laboratory without garlic bulbs.

For warehouse scale experiments, garlic bulbs of cv. ‘Morado’ and ‘Blanco’ were grown, processed and stored by Coopaman. VOCs were sampled from garlic stored in − 2 °C refrigerated warehouses at 3 month intervals. VOCs were collected onto TENAX/Sulficarb packed open ended thermal desorption tubes with a 1 cm diffusion pathway (Markes International, UK). Tubes were placed in open sided euro-pallet crates of garlic with one end cap removed and left to collect VOCs passively for 7 days before being recapped and shipped back to the laboratory for analysis.

TD tubes were desorbed on a TD100 thermal desorption system (Markes International Ltd., Llantrisant, Wales, UK), GC–MS data were processed using ChemStation (E.02.01.1177; Agilent Technologies Inc., Stockport, UK) and deconvolution and integration of signal peaks occurred in AMDIS (NIST, 2011), using a custom retention-indexed mass spectral library as previously described (Ludlow et al., 2021). MS spectra were compared to the NIST 2014 library (Mikaia et al., 2014) and only compounds scoring > 80% in forward and backward fit and retention index (RI ± 15) were included in the custom library as putatively identified. Known contaminants, VOCs not present in all three replicates of at least one sample, and those present in blank samples at > 1/10 of the mean abundance of all samples were excluded from further analysis. VOC data were normalised as a percentage of the total signal for each sample, and data were square root transformed to reduce bias towards highly abundant compounds in statistical tests.

### 2.6 Statistical analysis

GC-MS data were analysed in R Version 3.4.2 (R Core Team, 2019). Permutational multivariate analysis of variance (PerMANOVA) was performed to identify differences in the VOC profile associated with experimental parameters, using the function ‘adonis’ in the “vegan” package in R (Oksanen et al., 2019). Canonical analysis of principal coordinates discriminant analysis (CAPdiscrim) was performed in the “vegan” package in R to assess the significance of class discrimination according to experimental parameters (Legendre and Anderson, 1999, Anderson and Willis, 2003). Breiman's random forest algorithm was used for classification and regression in the “randomForest” package (R Core Team, 2019, Liaw and Wiener, 2002), and to identify components of the VOC profile which most significantly affected classification. Random forest analyses were plotted using Ordiplot in the “vegan” package and regressions were plotted using MDSplot in the “randomForest” package (Oksanen et al., 2019, Liaw and Wiener, 2002). Effects of cold stress on alliinase activity, water content and firmness were assessed using ANOVA.

## 3 Results

### 3.1 Cold stress

#### 3.1.1 Effects of cold stress on quality

**Fig. 1:**
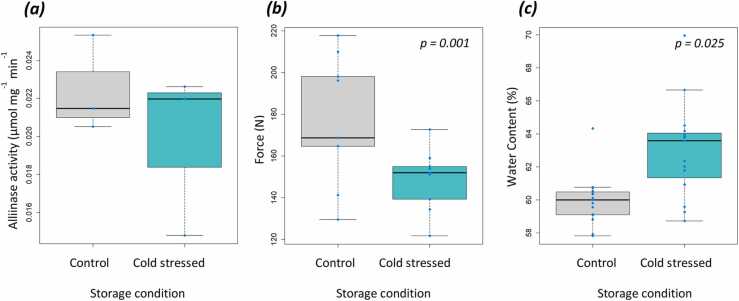
**:*****a)*** Alliinase activity (μmol mg^-1^ min^1^) of extracts from garlic (NS, *P* = 0.413, N = 3), ***b)*** water content of garlic cloves (FW-DW/FW) (*P* = 0.001, N = 6) and ***c)*** clove firmness, as measured by resistance to penetration of garlic cloves, following cold storage for 6 months at − 2 °C (control) and cold stressed for 96 h at − 5 °C (stress) (*P* = 0.025, N = 15). Different letters denote significance at *P* < 0.05 by ANOVA.

Alliinase activity in cold stressed garlic did not change significantly from the unstressed control (Fig. 1a). However, the water content of cloves was significantly higher in samples that were cold stressed, with the average water content increasing from 60.0% to 63.0% following cold stress (Fig. 1b). Furthermore, cold stressed cloves had a significantly lower firmness than unstressed cloves (Fig. 1c). The average force required to penetrate cloves fell from 18.0 kg cm^-1^ to 15.2 kg cm^-1^, with the firmest clove in the control group requiring 22.2 kg cm^-1^ for penetration and the weakest clove in the stressed group requiring just 12.4 kg cm^-1^.

#### 3.1.2 VOC profile of cold-stressed garlic

A total of 130 different VOCs were detected and 129 putatively identified across all samples. Alkanes (60) formed the major group followed by aromatics (15), aldehydes (9), alcohols and organosulphur (7 of each), esters and terpenes (6 of each), alkenes and ketones (5 of each), furans (4), carboxylic acids (3), phenol, alkenol and unknown (1 of each) (Supplementary Table S1A). Of the 130 compounds, 121 were detected in both − 2 °C stored and − 5 °C cold stressed samples, whereas nine compounds were detected in only one sample group (Table 1). VOCs putatively identified as dodecylcyclohexane, 2,6,10,14-tetramethylpentadecane and 6-methyltridecane were only detected in the − 5 ºC cold stressed samples. In terms of mean relative abundance, alkanes were the most highly abundant class of compounds, accounting for 46.6% of the total VOC profile. This was followed by alkenes (16.7%), aromatics (12.1%), ketones (5.5%), furans (5.3%), alcohols (3.5%), aldehydes (2.9%), organosulphur (2.3%), terpenes (1.7%), esters (1.4%), carboxylic acids (1.3%), alkenols (0.5%), phenols (0.2%) and unknown compounds (<0.1%) (Data in Supplementary Table S1B).

**Table 1:** list of compounds present in only one treatment group, in garlic cv. *Morado* stored for 6 months at − 2 °C or − 5 °C cold stressed. Relative abundance expressed as the average % of the total of all samples. *N* = 3.

Compound Number	Functional Group	Compound Name	Relative Abundance	
			-2 °C Stored	-5 °C Cold Stress
C8	Alkene	1-Octene	0.035 ± 0.00	ND
C44	Alkane	Methlycyclopentane	0.041 ± 0.01	ND
C59	Alkane	Dodecylcyclohexane	ND	0.011 ± 0.01
C65	Alkane	2,6-Dimethylheptadecane	0.408 ± 0.04	ND
C68	Alkane	2,5,5-Trimethylheptane	0.054 ± 0.01	ND
C81	Carboxylic acid	L-Lactic acid	0.492 ± 0.04	ND
C94	Terpene	p-Cymene	0.021 ± 0.01	ND
C97	Alkane	2,6,10,14-Tetramethylpentadecane	ND	0.240 ± 0.10
C121	Alkane	6-Methyltridecane	ND	0.172 ± 0.12

Values = average % abundance ± SD

**Fig. 2:**
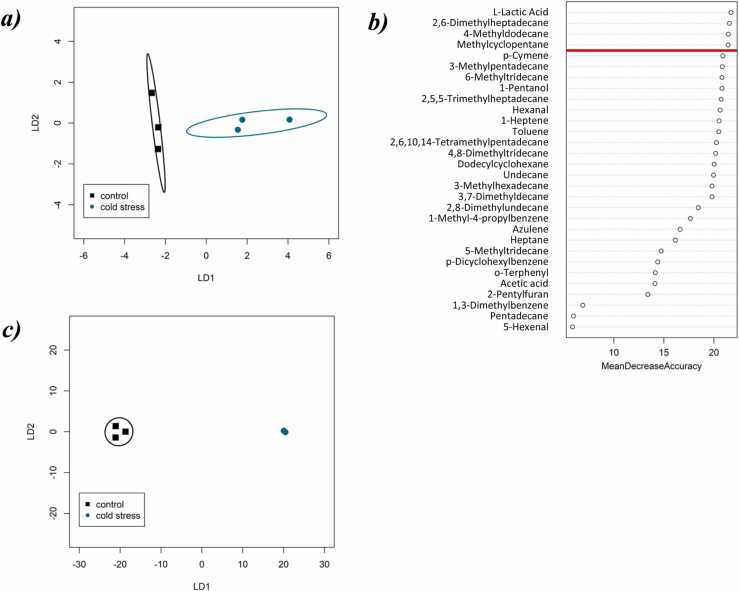
**:***a)* Ordination plot from CAP of the full VOC profile from garlic *cv. Morado* stored for 6 months at − 2 °C (black) and − 5 °C stressed (teal), each ellipse represents the 95% confidence interval (SD). The percentage of correct classification in the CAP model was 100% at a confidence of *P* = 0.05. *b)* significant features identified by random forest to predict cold stress in stored garlic bulbs. The compounds are ranked by the decrease in the model’s predictive accuracy from permuting the values in each feature. Red line denotes cut-off for inclusion in subsequent CAP model. *c)* Ordination plot from CAP of the top 4 compounds as identified by random forest from garlic *cv*. *Morado* stored for 6 months at − 2 °C (black) and − 5 °C stressed (teal), where each ellipse represents the 95% confidence interval (SD). The percentage of correct classification in the CAP model was 100% at a confidence of *P* = 0.05.

No significant differences were detected in the relative abundance of compound classes between – 2 °C and − 5 °C (*t*-Test, *P* > 0.05). Furthermore, VOC profiles of garlic did not change significantly overall in response to cold stress treatment (PerMANOVA; *P* = 0.10, R^2^ = 0.36). However, discrimination was achieved in CAP with an overall 100% classification success (Fig. 2a), and random forest identified compounds which had a high degree of discriminatory power between stressed and unstressed garlic (Fig. 2b).

In particular, four VOCs putatively identified as L-lactic acid (C81), 2,6-dimethylhetpadecane (C65), 4-methyldodecane (C58), and methylcyclopentane (C44) showed the highest mean decrease accuracy in the random forest algorithm (Fig. 2b). Of these four compounds, three were not detected in the − 5 °C cold stressed sample (Table 1), and one, the alkane 4-methyldodecane was present at below 5% of its average abundance in the − 2 °C stored samples (0.021 ± 0.004 in garlic stored at – 2 °C, and 0.001 ± 0.001 in cold stressed garlic). The relative abundance of these four VOCs showed an improved fit with the model (PerMANOVA; R^2^ = 0.99), and based on these compounds, discrimination was achieved in CAP with an overall classification success of 100% (Fig. 2c).

### 3.2 Warehouse trials of VOC analysis

A total of 141 VOCs were detected from crates of garlic stored in − 2 °C refrigerated warehouses, of which 139 were putatively identified across all samples, including all three time points (after 0, 3 and 6 months of cold storage) and both cultivars: ‘Morado’ and ‘Blanco’ (Supplementary Table 2 A). Here, alkanes were the most prevalent class of compounds (31), followed by aromatic and organosulphur compounds (19 each), aldehydes (16), alcohols and ketones (12 each), terpenes (8), alkenes and esters (5 each), furans (4), carboxylic acids (3), ethers and unknown compounds (2 each), and nitrogenous, oxiranes and phenols (1 each). In terms of relative abundance (Supplementary Table S2B), organosulphur compounds made up the largest proportion of the VOC profile of warehouse stored garlic, accounting for an average of 45% of the total across all samples. The next most abundant class of compounds was alcohols (14.8%), followed by aldehydes (9.6%), aromatic compounds (7.0%) and ketones (6.1%) (Supplementary Table S2C). Significant differences in relative abundance were detected across storage times in all classes except carboxylic acids, ethers and oxiranes, and the majority of changes were between 3 m and 6 m of storage.

**Fig. 3:**
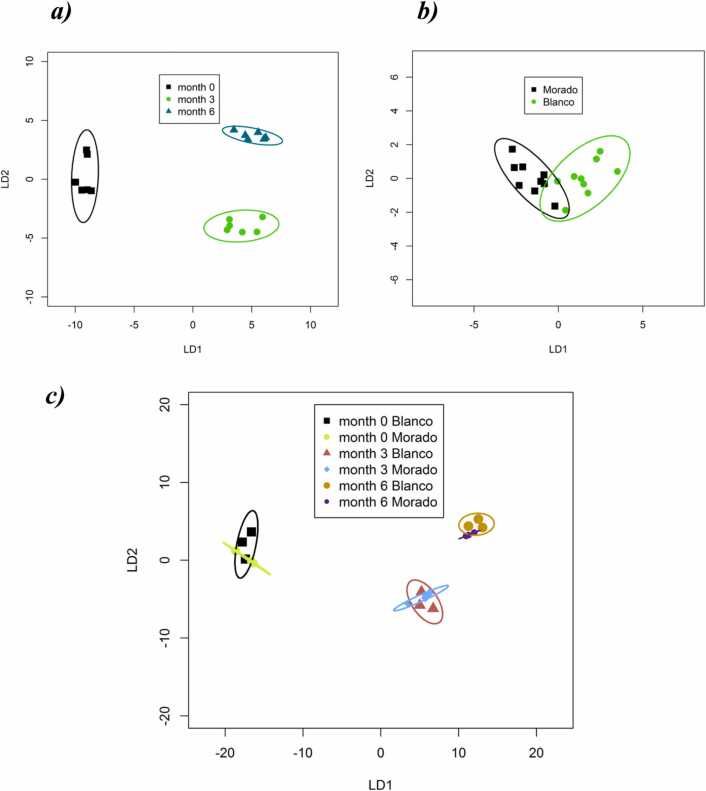
**:** Ordination plot from Canonical Analysis of Principal Ordinates (CAP) based on all VOCs from garlic *cv*. *Blanco* and *Morado* using TD-GC-ToF-MS. CAP models were produced for warehouse stored garlic VOCs, which considered (***a***) storage time (N = 6), (***b***) cultivar (N = 9) and (***c***) both storage time and cultivar (N = 3). The plots use the first two linear discriminants of the CAP model and each ellipse represents the 95% confidence interval (SD). The percentage of correct classification in the CAP model was 100% at a confidence of *P* = 0.05.

Whole VOC profiles varied significantly over time across both cultivars (PerMANOVA, *P=* 0.0001, *R*^*2*^ = 0.608). However, VOCs did not change significantly between cultivars (PerMANOVA *P=* 0.175, *R*^*2*^ *= 0.036*). Similarly, the whole VOC profile discriminated over time using CAP (Fig. 3a & c) but not between cultivars (Fig. 3b & c).

**Fig. 4:**
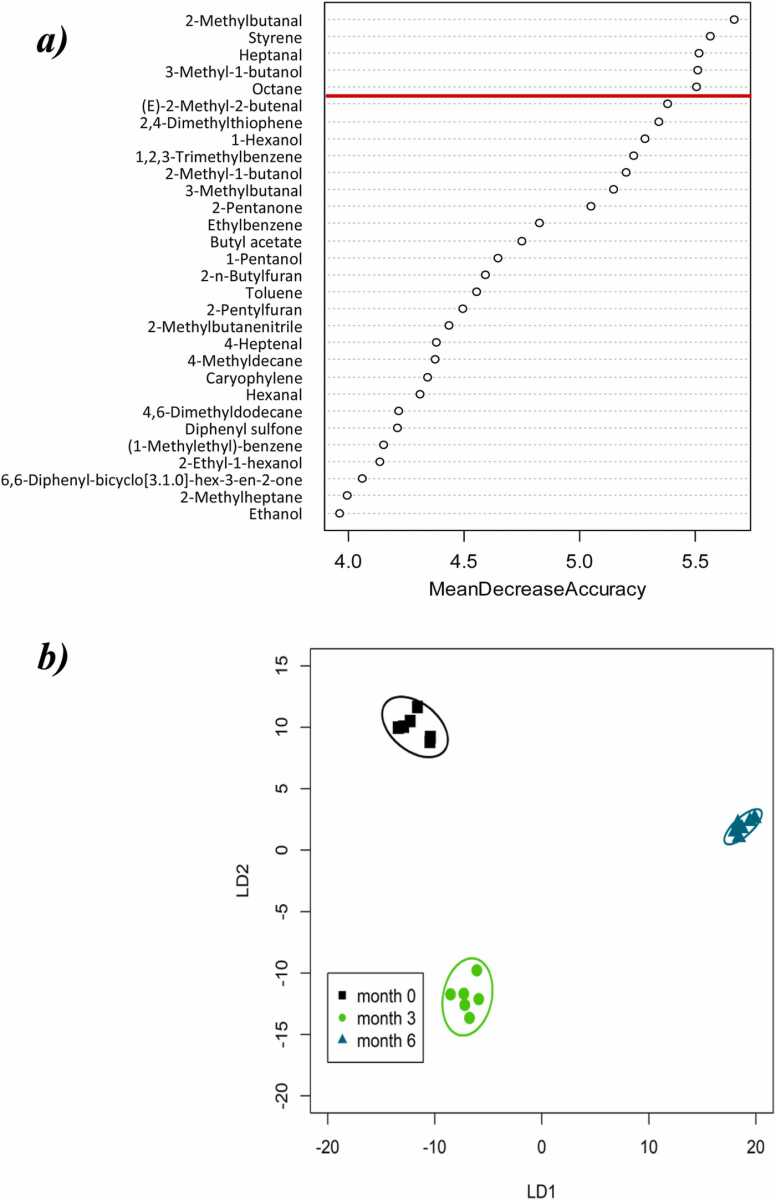
***a***) significant features identified by random forest classification to predict storage time in warehouse stored garlic bulbs. The compounds are ranked by the decrease in the model’s predictive accuracy from permuting the values in each feature. Red line denotes cut-off for inclusion in subsequent CAP model. ***b***) ordination plot from CAP of the five VOCs with highest predictive accuracy according to random forest for storage time. The plots use the first two linear discriminants of the CAP model and each ellipse represents the 95% confidence interval (SD). The percentage of correct classification in the CAP model was 100% at a confidence of *P* = 0.05. N = 3.

The VOCs which allowed the best discrimination between storage time in the warehouse were identified using a random forest classification (Fig. 4a), and discrimination between the two groups was achieved with CAP using just five VOCs, with a 100% correct classification (Fig. 4b). Furthermore, reanalysis by PerMANOVA showed an increase in the R^2^ value for storage time to 0.981, showing a greatly improved fit of the model to the data and suggesting the abundance of these VOCs is indeed closely associated with storage time. These five VOCs belonged to a diverse range of chemical groups and included two aldehydes, and one each of alkanes, alkenes, and alcohols (Fig. 4a).

## 4 Discussion

A brief period of storage at − 5 °C affected both water content and firmness of the garlic bulbs, but not alliinase activity. The water content for both samples was within the expected range for the water content of garlic at 6 months of − 2 °C storage (Pardo et al., 2007) indicating that the storage conditions were in line with normal practice. Variation in water content has been noted previously across samples stored at different sub-zero temperatures (James et al., 2009) and may be an effect of the temperature treatment. The reduction in firmness, may also be a consequence of the cold stress treatment. Loss of firmness during storage has been associated with sprouting at higher temperatures (Vazquez-Barrios et al., 2006), and differences in temperature between 0 and − 3 °C affected firmness as assessed in sensory studies (Volk et al., 2004). The exposure to low temperature may accelerate middle lamella degradation, noted in onion with storage (Coolong et al., 2008) or be related to a loss of cellular turgidity due to cellular damage. The lack of an effect on alliinase activity is of interest to the industry indicating that although the firmness and water content changes indicate early signs of damage, this short period of cold stress may be considered safe in the context of garlic quality. However, it is possible that longer or repeated periods of − 5 °C may affect alliinase activity maintenance over time.

The VOC profile of fresh garlic and other *Alliums* changes in response to preharvest factors such as soil composition (Abbey et al., 2004), water deficit (Abbey et al., 2003), pathogen attack (Li et al., 2011, Vikram et al., 2005) and attack by nematodes (Matsumoto et al., 2020). Postharvest, storage time (Lawson et al., 1991, Ludlow et al., 2021) and treatments such as bulb irradiation have also been shown to change the VOC profile (Wu et al., 1996). Crushing and heating the garlic also has profound effects on the aroma profile (Abe et al., 2020). However, the effects of short-term cold stress were previously unexplored. The abundance of key sulphur compounds, which are the main determinants of the scent of garlic (Mazza et al., 1992) were not affected. In addition, the overall abundance of VOC classes did not change, reflecting the subtlety of the changes. The extent of the variability across replicates is likely due to differences in the physiology of the individual bulbs. Over 60 compounds were detected which were previously not reported in untreated garlic (Abe et al., 2020, Ludlow et al., 2021), although only two additional VOCs were detected here following the cold stress, compared to the garlic held under standard chilled condition of − 2 ºC suggesting that many of the extra VOCs detected may be due to increased sensitivity of the detection here or the different variety studied.

Little is known about cold stress responses in garlic between two sub-zero temperatures, especially over such a short induction period. However, temperatures as relatively warm as 5 °C have been shown to increase the synthesis of phenolic compounds and anthocyanins in garlic, compared to a 23 °C control, indicating that the bulb does respond to cold stress (Dufoo-Hurtado et al., 2015). The four most highly predictive compounds for − 5 °C cold stress were putatively identified as L-lactic acid, 2,6-dimethylheptadecane, 4-methyldodecane and methylcyclopentane, although unequivocal identification of these compounds would require further work. 2,6-dimethylheptadecane has not previously been reported in garlic to the best of our knowledge but is found in plants including *Euclea crispa* (Palanisamy and Ashafa, 2019) and *Adiantum flabellulatum* (Kang et al., 2009). Similarly, 4-methyldodecane is novel in garlic, but reported in *Cuscuta reflexa* (Afrin et al., 2019), *Catha edulis* (Alsanosy et al., 2020) and *Oryza sativa* L. (Xia and Li, 2018). Methylcyclopentane has been reported in *Trifolium pratense* as the second most abundant compound in methanolic leaf extracts (Akbaribazm et al., 2020) and again as a major component of *Brassica juncea* petroleum ether extract (Aziz et al., 2020). It is possible that these compounds are produced by the garlic in response to the stress treatment as a defence response, or that the softening is associated with reduced cell integrity and the potential for bringing new enzymes and substrates together which in intact cells are normally compartmentalised.

Warehouse trialling of the GCMS based method for VOC analysis successfully discriminated garlic stored for different periods of time. Interestingly, the relative abundance of some VOCs fluctuated over the 6 m period. Garlic bulbs are a complex matrix and although the cold storage regime reduces sprouting, after long periods some changes in development may occur as well as changes in microflora. These changes may result in effects on the garlic metabolism that are non-linear over time. For example, fluctuation in alliinase extract was previously noted over 6 m of cold storage, but not when the bulbs were stored at ambient temperature (Ludlow et al., 2021).

The VOCs did not discriminate between cultivars. This may be due to the sampling method as both cultivars were stored in the same warehouse and the VOCs would have mixed in the air. However, this could be considered a benefit, as the main objectives of such a tool would be to assess freshness, not discriminate between cultivars. The passive sampling method detected a similar number of compounds, with 141 VOCs reported here versus 150 detected previously (Ludlow et al., 2021) using similar techniques. However, only 48 were found in both studies. The volatilome is complex and is a product of all the interactions between the garlic and its environment, thus differences could be due to the varietal or environmental affects between lab and warehouse storage conditions. Although it might be possible to consider only this shared set of 48 VOCs this would risk losing informative VOCs from the warehouse experiment.

Only 15 VOCs were qualitatively different between the 0 and 6 m time points, indicating that the majority of changes in the volatilome are quantitative. This is perhaps not unexpected in a warehouse scale experiment where the VOC signal will be an integral of all the bulbs in the crate. The five most highly predictive compounds for storage time in warehouse stored garlic VOCs were putatively identified as 2-methylbutanal, styrene, heptanal, 3-methyl-1-butanol and octane. Styrene and 3-methyl-1butanol have previously been reported in stored onions, but only in the presence of *Fusarium oxysporum* f. sp. *Cepae* (Wang et al., 2019), which may suggest that there is a component of the VOC profile which arises from postharvest spoilage organisms and which is highly predictive of storage time. Besides infecting onions, *Fusarium oxysporum* f. sp. *Cepae* is also one of the most serious pathogens of garlic, responsible for basal rot disease (Chand et al., 2017). The compound 2-methylbutanal has previously been identified in the VOCs of black garlic (Molina-Calle et al., 2017), and the headspace of *Allium tenuissimum* L. flowers (Zhang et al., 2021). It was also associated with seed deterioration (Colville et al., 2012), but to our knowledge has not previously been associated with storage or garlic bulbs. Of 17 VOCs whose relative abundance previously changed during lab-based garlic storage experiments (Ludlow et al., 2021), three were detected here in the warehouse experiment: putatively identified as limonene, benzeneacetaldehyde and 3-carene. However, none were identified as having high predictive accuracy of storage time in the warehouse, indicating that scaling up to warehouse level is necessary to identify appropriate markers for the industry.

In conclusion, VOCs have potential as markers for garlic storage assessment even in an industrial setting where cultivars are stored together. However, different VOC markers are needed for detecting changes during optimal storage and breaches in the cold storage regime, multiple markers may be needed, and markers for cold stress would need further verification at warehouse level.

## Funding sources

This work was supported by the Biotechnology and Biological Sciences Research Council/Knowledge Transfer Network, UK (Grant Reference: BB/M014967/1) , and Neem Biotech Ltd, UK (iCASE PhD studentship for RAL).

## CRediT authorship contribution statement

**Richard A. Ludlow:** Investigation, Writing – original draft. **Gareth Evans:** Conceptualization**. Michael Graz:** Conceptualization**. Gracia Marti:** Methodology**. Puri Castillo Martínez:** Investigation. **Hilary J. Rogers:** Conceptualization**,** Funding acquisition, Writing – review & editing, Supervision. **Carsten T. Müller:** Conceptualization, Supervision, Formal analysis, Writing – review & editing.

## Declaration of Competing Interest

The authors declare that they have no known competing financial interests or personal relationships that could have appeared to influence the work reported in this paper.
